# Cadaveric study validating in vitro monitoring techniques to measure the failure mechanism of glenoid implants against clinical CT

**DOI:** 10.1002/jor.23899

**Published:** 2018-04-25

**Authors:** Sarah Junaid, Thomas Gregory, Shirley Fetherston, Roger Emery, Andrew A Amis, Ulrich Hansen

**Affiliations:** ^1^ Mechanical Engineering and Design Aston University Birmingham B4 7ET United Kingdom; ^2^ Department of Mechanical Engineering Imperial College London London SW7 2AZ United Kingdom; ^3^ Service de Chirurgie Orthopedique et Traumatologique Université Paris Descartes, Hôpital Européen Georges Pompidou Paris France; ^4^ Radiology, St Mary's Hospital London W2 1NY United Kingdom; ^5^ Musculoskeletal Surgery Imperial College London London W6 8RF United Kingdom

**Keywords:** glenoid loosening, fixation failure, CT, radiolucent lines

## Abstract

Definite glenoid implant loosening is identifiable on radiographs, however, identifying early loosening still eludes clinicians. Methods to monitor glenoid loosening in vitro have not been validated to clinical imaging. This study investigates the correlation between in vitro measures and CT images. Ten cadaveric scapulae were implanted with a pegged glenoid implant and fatigue tested to failure. Each scapulae were cyclically loaded superiorly and CT scanned every 20,000 cycles until failure to monitor progressive radiolucent lines. Superior and inferior rim displacements were also measured. A finite element (FE) model of one scapula was used to analyze the interfacial stresses at the implant/cement and cement/bone interfaces. All ten implants failed inferiorly at the implant‐cement interface, two also failed at the cement‐bone interface inferiorly, and three showed superior failure. Failure occurred at of 80,966 ± 53,729 (mean ± SD) cycles. CT scans confirmed failure of the fixation, and in most cases, was observed either before or with visual failure. Significant correlations were found between inferior rim displacement, vertical head displacement and failure of the glenoid implant. The FE model showed peak tensile stresses inferiorly and high compressive stresses superiorly, corroborating experimental findings. In vitro monitoring methods correlated to failure progression in clinical CT images possibly indicating its capacity to detect loosening earlier for earlier clinical intervention if needed. Its use in detecting failure non‐destructively for implant development and testing is also valuable. The study highlights failure at the implant‐cement interface and early signs of failure are identifiable in CT images. © 2018 The Authors. *Journal of Orthopaedic Research* ^®^ Published by Wiley Periodicals, Inc. on behalf of the Orthopaedic Research Society. J Orthop Res 36:2524–2532, 2018.

A study investigating total shoulder arthroplasty outcomes (TSA) found loosening to be the most common complication.^1^, ^2^ This has been confirmed by other recent studies^3^, ^4^ and has accounted for up to 44% of glenoid implant failures.^5^ In clinical and cadaveric studies on glenoid fixation, the absence of visual observation requires investigators to depend on the presence of radiolucent lines in radiographs and clinical examination to judge the quality of the implant fixation. Clinically the majority of radiolucent lines have been identified in the inferior region of the implant, possibly indicating glenoid loosening and a mechanical weakness inferiorly.^6^, ^7^, ^8^ Radiographs are fairly accurate when identifying advanced stages of loosening, which is defined by a visible shift of the implant or a radiolucent line encompassing the entire implant fixation, commonly referred to as “definitely loose.”^5^ However, early loosening stages are ambiguous in radiographs and impossible to define accurately. Even when identifying definite loosening, a study on failed TSA found 85% of retrieved glenoid implants that were definitely loose were identified from the radiographs,^9^ which indicates an under estimation of the loosening problem.

In vitro studies have attempted to quantify glenoid loosening by measuring the horizontal rim displacement during superior‐inferior cyclic rim loading of the glenoid implant.^10^, ^11^, ^12^ These fatigue studies, which use bone substitute foam to eliminate the effect of bone variability, found a positive correlation between inferior rim displacement and number of cycles. However, the disadvantage in using this quantitative method is that these studies were not able to visualize failure progression of the embedded glenoid. Therefore it is difficult to link any quantitative data to actual failure. This gap has been addressed in two in vitro 2D studies correlating failure progression with both rim displacement and head displacement.^13^, ^14^ The latter study allowed direct observation of the implant fixation, and found a correlation between inferior fixation failure and superior and inferior rim displacements.^14^ The idea of using head displacement to monitor failure progression was also introduced.

A significant drawback to these in vitro studies is that clinical measurement methods such as radiographs were not used to correlate their quantitative findings. In response to this, a study using implants embedded in bone substitute investigated CT imaging to monitor early stages of fixation failure.^15^ The study found a correlation between radiolucent lines in the final CT images and implant‐cement interface fixation failure from sectioning the specimens. The main drawback was the use of bone substitute, which allowed the displacement correlation to be identified but does not directly represent the human glenoid bone structure, which is structurally heterogeneous, highly variable and therefore can have variable bone‐cement interfacial strengths.

In vitro testing of glenoid loosening has attempted to quantify or monitor fixation failure through rim displacements, head displacements, and CT imaging. However, there is a lack of clarity on how these measures correlate to actual failure or failure progression. Comparing these findings to the clinical setting is also limited due to lack of cadaveric testing. This cadaveric study aims to identify any correlations between in vitro monitoring methods and clinical methods to measure glenoid prosthesis failure.

## MATERIALS AND METHODS

Eleven fresh‐frozen cadaveric scapulae were used, with ethics committee approval. One was excluded due to very poor sclerotic bone. Another was defined as partially sclerotic, but was included in the study, resulting in a total of ten scapulae that were implanted and tested.

### Monitoring Methods

Three methods were used to observe and monitor failure progression; quantitative in vitro measures, qualitative in vitro observations and clinical observations. These will be referred to as quantitative, qualitative, and clinical for the rest of the paper. Quantitative measures used were superior and inferior rim displacements as specified by the ASTM testing standard (F2028‐17^16^) and vertical head displacement changes. The qualitative measures used were visual observation during testing and cross‐sectional observation under microscopy post‐testing. Finally, the clinical measure used was radiolucent lines in CT images of the specimens. Correlations were sought between the qualitative and quantitative measures and the clinical observations.

### Specimen Preparation

**Figure 1:**
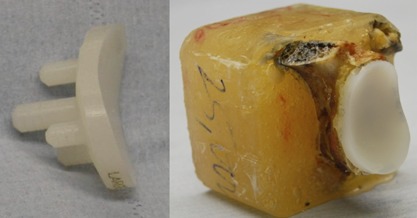
Curved‐back cemented glenoid implant (left). Cadaveric scapula cemented and potted for testing (right).

**Figure 2:**
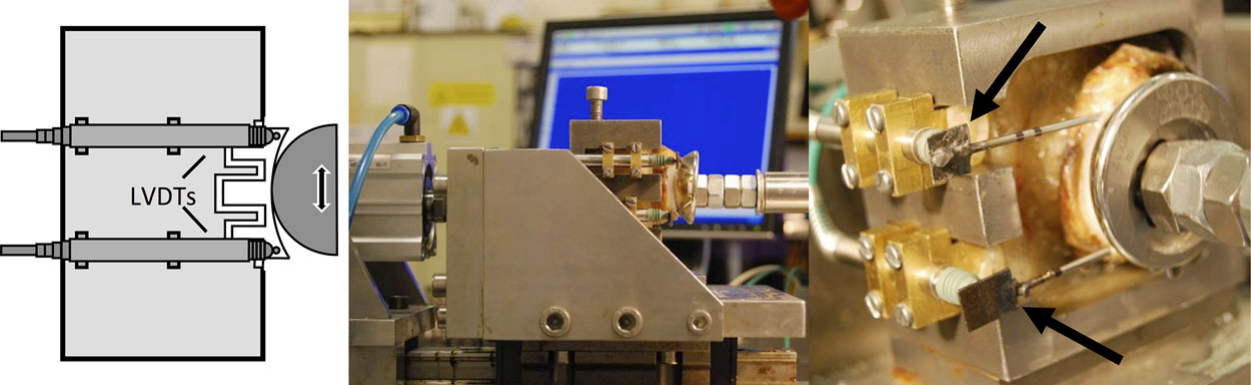
Mechanical glenoid fixation loosening cadaveric test (middle), which is also shown as a schematic (left). Reference pins (arrows) were used to monitor horizontal rim displacement using LVDTs attached directly to the specimens (right).

The ten scapulae were implanted with a commercially available glenoid implant, an Aequalis all‐polyethylene, curved‐back, pegged design (Tornier Inc., Grenoble, France) (Fig. 1). Three small, six medium and one large glenoid with radial curvatures of 27.5, 30, and 32.5 mm, respectively, were implanted by an experienced shoulder surgeon (T.G.). The soft tissue and labrum were excised. The glenoid surface was reamed, removing the cartilage layer, and care was taken to maintain the subchondral layer. The glenoid implants were cemented using Simplex® bone cement (Stryker Europe, Montreux, Switzerland). The scapulae were cut to size using an Exakt 310 CP diamond‐tipped high precision saw (Exakt Technologies Inc., Oklahoma City) and cemented using Simplex® bone cement into the specimen holder. Care was taken to ensure the correct seating of the glenoid component with no tilt (Fig. 1). Two holes were drilled into each glenoid implant to accommodate a 2 mm diameter rod at the superior and inferior edge of the glenoid, 2.5 mm from the corresponding rim. The two rods were prepared as reference points to measure the corresponding rim displacements via two displacement transducers (LVDTs) (Fig. 2).

### Mechanical Test

The scapulae were cemented into the specimen holder and tested using a testing rig compliant to the ASTM standard F2808‐17.^16^ A compressive horizontal load of 750 N was applied throughout. A 24 mm humeral head manufactured by the implant company was used to articulate onto the implants for all specimens. Thus, the three glenoid sizes; small, medium and large, corresponded to a radial mismatch of 3.5, 6, and 8.5 mm, respectively. The specimens were tested without a water bath at room temperature, and the scapulae and joints were kept wet via a water spray. LVDTs were attached directly to the specimen and horizontally aligned to measure horizontal rim displacement at the superior and inferior rim via reference pins inserted at the implant rim edge as specified by the standard^16^ (Fig. 2). The rim displacements were measured every 2000 cycles without stopping the test with visual failure as the primary outcome measure. Every 4000 cycles the vertical head displacement was readjusted to maintain the testing loads.

The loading regime was derived from the subluxation curves of two medium glenoid prostheses implanted in bone substitute. The vertical load was chosen to be 400 N by deriving 90% of the subluxation load. A common load was used throughout, despite testing 3 different implant sizes. The subluxation load differences between large and medium glenoid prostheses were comparable at 500 N and 465 N respectively. Thus a standardized loading of 400 N was used for all specimens.

### CT Scans

CT scans were taken of all the scapulae before implantation, after implantation, at 20,000, 40,000, 60,000 cycles and after failure or at 200 000 cycles if failure did not occur.

**Figure 3:**
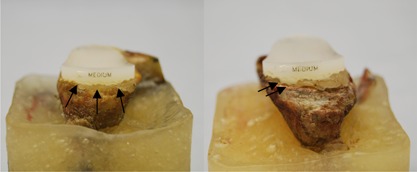
Failure definition: “partial failure” as inferior rim distracts away from cement (left) and “failure” as inferior pegs visible (right).

**Figure 4:**
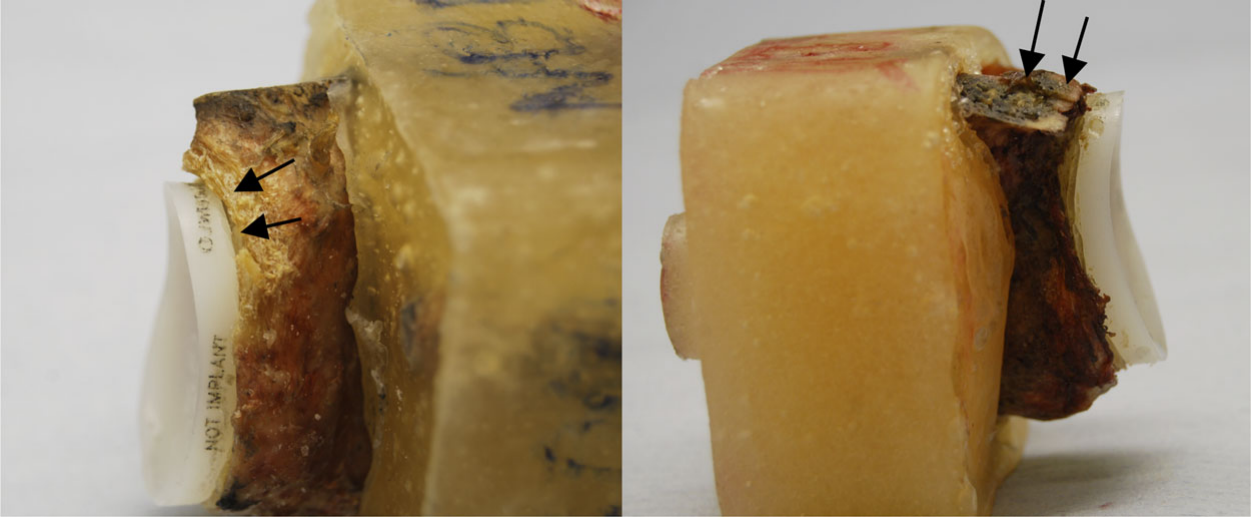
Superior failure‐rim distracts away from cement (left) and bone crushing‐bone fracture or implant embedding (right).

During testing, failure visually was defined in two stages (Fig. 3), initial failure was indicated by visible distraction of the inferior glenoid rim from the cement or bone substitute block. Partial failure was defined as the point when the inferior pegs were visible during inferior rim distraction, where the test was stopped. Partial failure is referred to in the following text as failure. Superior bone crushing was defined by visible embedding of the superior implant rim or bone fracture and superior failure was defined as visible distraction of the superior rim. “CT partial failure” was defined as a radiolucent line between the implant rim and the cement and the bone or between the cement and bone (Fig. 4). “Complete failure” was defined as a radiolucent line reaching the inferior pegs in the CT images. Microscopic images were compared to the final CT image.

### Post‐Testing Observations

After testing to failure or to 200,000 cycles, the specimens were sectioned through the superior‐inferior centerline using an Exakt 310 CP diamond‐tipped saw (Exakt Technologies Inc.) and the fixation and bone conditions were observed under a Nikon SMZ 800 microscope (Nikon Instruments Inc., New York) with a magnification of ×20.

Statistical significance between the rim displacement measures and correlation to visual failure as well as vertical head displacement to visual failure were tested using a single factor ANOVA tests.

### Finite Element Modeling

A three‐dimensional finite element (FE) model was constructed using a CT scan of one of the scapulae. Amira® (Visage Imaging, San Diego, CA) was used to construct the tetrahedral mesh using over 100,000 elements and the glenoid implant model acquired from the implant company (Tornier Inc., Grenoble, France) was inserted into the bone model. Marc/Mentat 2001 (MSC Software Corporation, CA) was used to perform the FE analysis. The material properties of the bone were assigned using an in‐house program.^17^ The Carter and Hayes^18^ relation was used to describe the material properties of bone from the CT number: E = 2875ρ_app_^3^, where E is the Young's modulus, ρ_app_ is the apparent density and CT numbers 30–2000 correspond to densities 0.3–1.8 g/cm^3^ on a linear scale. The strength of the cancellous bone was calculated using the following relationship: S = 51.58 ρ^2^ using the lowest density value in the bone image of 0.3 g/cm^3^.^18^ PMMA Bone cement was given a Young's modulus of 2.2 GPa and Poisson's ratio of 0.3.^19^

The contact surfaces were bonded and the humeral head was modelled as a rigid hemisphere. The scapula was cut to size, as in the in vitro test. The surface nodes of the scapula beyond the scapula neck were constrained in all three axes. The frictional coefficient between the humeral head and glenoid was 0.07.^11^ A compressive load of 750 N was applied to the humeral head and a vertical load was applied via head displacement of 11 mm, to generate a load/displacement subluxation curve. The FE mesh was tested to load convergence.

## RESULTS

### Qualitative Measurement Results

**Figure 5:**
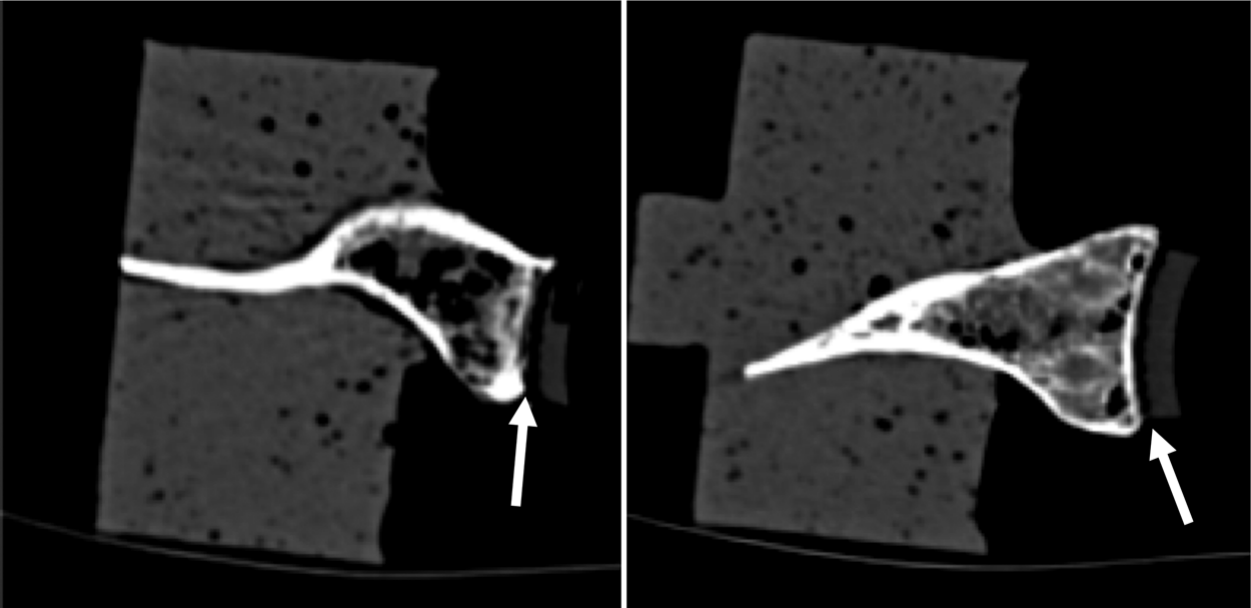
CT slices of the transverse plane showing an example of superior (left) and inferior (right) failureat the implant‐cement interface in a specimen.

All ten implants visibly failed except one, which only partially failed (Fig. 3) where the test stopped after 200,000 cycles. Six failed exclusively at the implant‐cement interface, two failed both at the implant‐cement and cement‐bone interface and two failed superiorly due to cortical bone failure (Fig. 4). Implant failure occurred between 16,300 and 122,500 cycles, with a mean (± SD) of 80,966 ± 53,729 cycles. The earliest specimen to fail had previously been identified as partially sclerotic. The partially failed implant was stopped at 200,000 cycles, although some superior and inferior implant‐cement distraction was observed and CT scans revealed initial good implant seating. All final CT scans confirmed failure, which were observed visually (Fig. 5), however, in three specimens it was difficult to identify which interface loosening was apparent either visually or with CT. No significant difference was found between the three radial mismatches with respect to cycles to failure.

**Figure 6:**
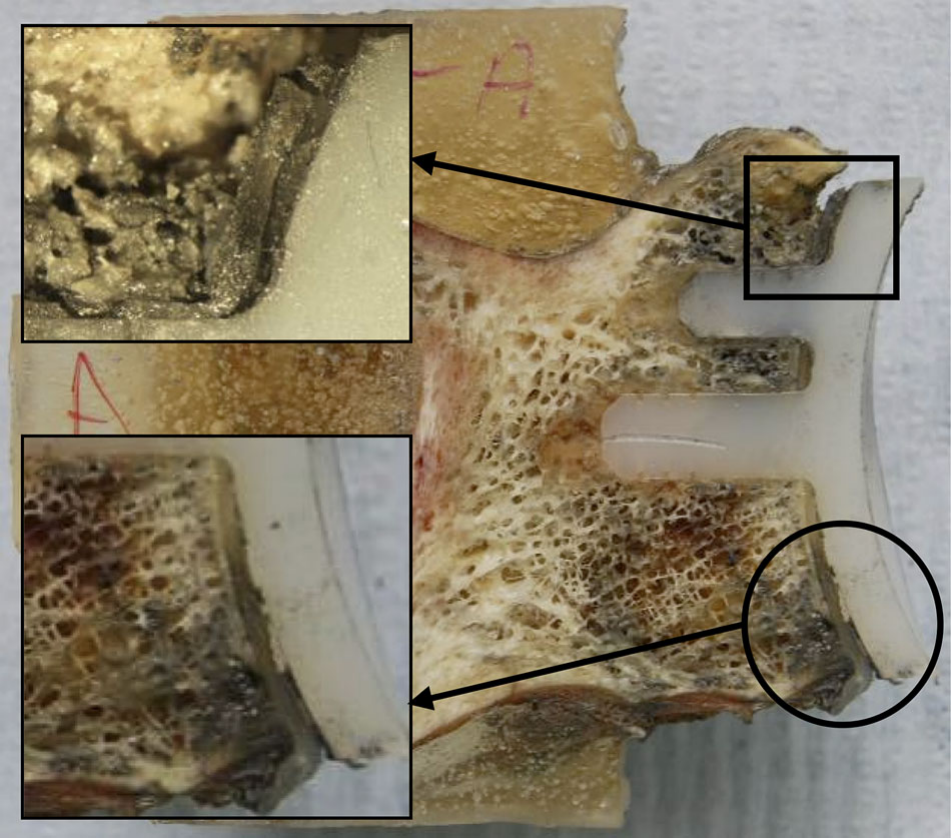
Cross‐sectional slice of same specimen as CT after failure. Note: inferior failure of the implantcement(circle) and superior bone crushing (square).

The visual examination of the sectioned specimens confirmed clear failure at the implant‐cement interface and superior bone crushing, as was observed from inspection of unsectioned specimens (Fig. 6). The microscopic study revealed the cement thickness varied from 0.5 to 1.5 mm and was cracked in three specimens at one of the peg junctions where bending stresses had been experienced. There were no other apparent cement fractures anywhere else. In one case, the implant completely detached at the implant‐cement interface, the cement embedded in the peg grooves was still intact.

### Quantitative Measurement Results

**Figure 7:**
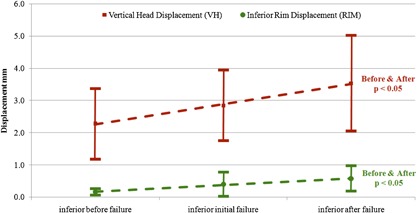
Positive correlation between vertical head displacement (*p* < 0.05) and inferior rim displacement(*p* < 0.05) with visual failure.

Inferior rim displacement and vertical head displacement both increased with observed failure (Fig. 7). The positive correlation between vertical head displacement before failure and at failure was statistically significant (*p* < 0.05). This was also true for the inferior rim displacement (*p* < 0.05). The mean vertical head displacement (± SD) before and after failure was 2.3 ± 1.1 and 3.5 ± 1.5 mm, respectively.

### Clinical Measurement Results

**Figure 8:**
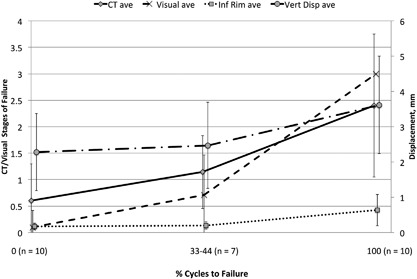
A plot of the mean clinical CT failure* and qualitative visual measures* with quantitative inferiorrim and vertical head displacement against cycles to failure, normalised to a percentage. The plot showscorrelation between displacements with CT and visual failure. *No failure, 1; partial failure, 2; and failure, 3.

All four failure measures: Clinical CT, qualitative visual, quantitative vertical head, and quantitative inferior rim displacement, positively correlated with cycles to failure (Fig. 8 and Table 1). On average, failure was identified in clinical CT images before visual failure was observed (Fig. 8). This observation was found in 8/10 shoulders. In the remaining two shoulders, CT and visual failure were observed together in one and visual failure observed first in the other.

**Table 1:** Tabulated Form of Figure 8 Showing Comparison of Percentage Cycles to Failure at no Failure (0%), Partial Failure (33–44%) and Failure (100%) Compared to the Four Failure Measures; Clinical CT Failure, Qualitative Visual Failure, Quantitative Inferior Rim Displacement and Quantitative Vertical Head Displacement, Respectively

	CT Failure^a^		Visual Failure^a^		Inferior Rim Displacement (mm)		Vertical Head Displacement (mm)	
% Cycles to Failure	Mean	SD	Mean	SD	Mean	SD	Mean	SD
0% (*n* = 10)	0.6	0.7	0.1	0.3	0.2	0.1	2.3	1.1
33–44% (*n* = 7)^b^	1.1	0.7	0.7	0.8	0.2	0.1	2.5	1.2
100% (*n* = 10)	2.4	1.3	3.0	0.0	0.6	0.4	3.6	1.4

^a^ No failure, 1; partial failure, 2; and failure, 3.

^b^ Three implants failed before partial failure was captured.

Quantitatively, mean vertical head displacement increased with cycles to failure, visual failure and CT failure in all ten specimens. On average inferior rim displacement did not change at 33–44% cycles to failure (partial failure stage) until 100% failure occurred. Furthermore, the inferior rim displacement fluctuated throughout testing compared to vertical head displacement, which progressively increased.

### Finite Element Modeling

The implant/cement interface normal stress predicted by the FE model showed superior compressive stresses and inferior tensile stresses. Tensile peak stresses were found at the base of the pegs (2.5 MPa) and peaked at the inferior edge of the implant (1 MPa).

**Figure 9:**
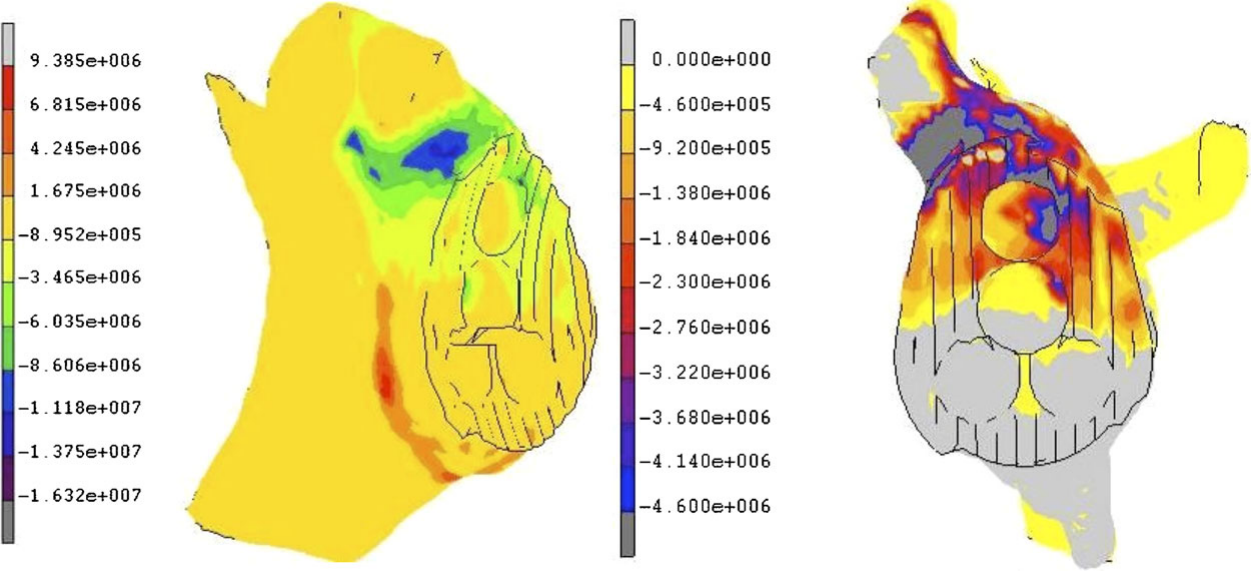
Color plot of the cadaveric bone showing minimum principal stress (compressive stresses‐blue) and maximum principal stress (tensile stresses‐red) (left). Color plot of minimal principal stress (compressive only) showing dark gray areas exceeding 4.6 MPa (predicted bone strength) (right).

The strength of the cancellous bone was calculated using the lowest bone density as 4.6 MPa, the compressive stresses in the bone exceeded this superiorly during loading, corroborating the experimental finding of superior bone crushing (Fig. 9).

## DISCUSSION

The most important finding of this study was the significant correlations found between three laboratory‐based qualitative and quantitative measures of glenoid loosening (visual failure, inferior rim displacement, and vertical head displacement) and clinical CT images of loosening. Using the ASTM F2028‐17^16^ testing method allowed a standardized and repeatable method of mechanically testing the integrity of the glenoid prosthesis/cement/bone interface. For this study, the standard was used to test glenoid fixations in cadaveric bone rather than bone substitute. The advantage of quantifying failure is that it serves as a comparative measure between implant designs and allows for controlled testing of various surgical conditions such as poor bone quality, cement interdigitation and bone wetness. From the three measurements used in this study visual failure was the surest way of identifying failure, however, it is subjective and labor intensive. Inferior rim displacement is not subjective but requires alteration of the implant by drilling or fixing a measuring platform to the rim. Finally the head displacement does not require any alterations to the test or additional measuring equipment, however, requires load‐controlled testing. All three measures had previously not been directly compared to what is observed clinically using cadaveric bone. This study has shown that what may be seen in clinical CT imaging correlated with detailed measurements of loosening phenomena on the specimens.

**Figure 10:**
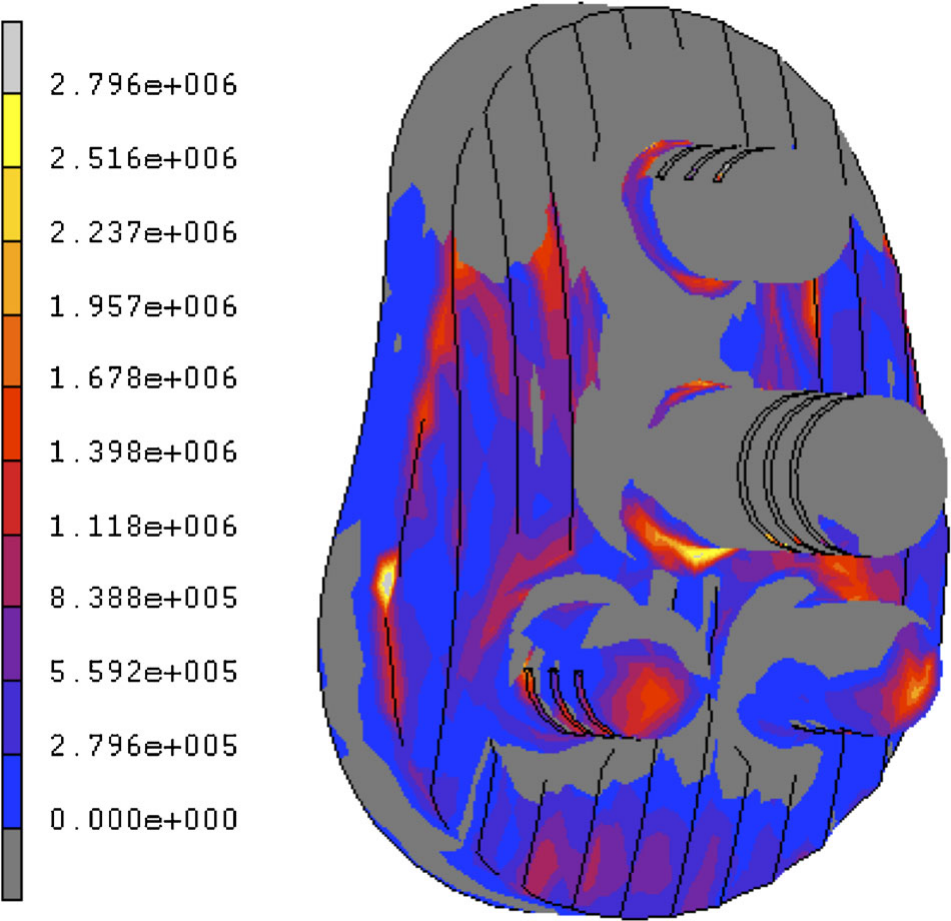
Tensile normal contact stresses at the implant/cement interface. Note: peak stresses at the inferioredge and pegs reaching up to 1 and 2.5 MPa, respectively.

Although the sample size was small, all ten cadaveric scapulae tested failed inferiorly at the implant‐cement interface, and two of these also failed at the cement‐bone interface. No specimen failed at the cement‐bone interface alone, however, superior bone crushing was also observed clearly in three specimens. The CT scans indicated failure at the observed interface in seven cases and was able to detect failure before or with visual failure in nine specimens. All in vitro measurements correlated with CT failure, with quantitative rim displacement and head displacement both showing a significant increase from no failure to failure (*p* < 0.05) (Fig. 7). The FE model showed peak tensile loads at the inferior rim and at the base of the inferior pegs. Unpublished work in our laboratory have found implant/cement interface strengths at between 0 and 1 MPa for smooth implant surfaces, placing the peak tensile stresses within the failure range, thus possibly corroborating the experimental findings (Fig. 10).

Furthermore, the 4.6 MPa predicted bone failure compressive strength was exceeded during the tests. This prediction was corroborated by the superior crushing found in seven of the ten experimental samples.

Clinical results have indicated predominantly cement‐bone failure via radiographic examination. This study has investigated this phenomenon using a standardized in vitro cyclic test, post‐testing microscopic evaluation and monitoring failure both visually and quantitatively. The question of where the fixation is weakest is not a simple one, considering implant roughness, cement interdigitation, cement thickness, wetness of the bone and bone quality all contribute to the interfacial conditions. Using a smooth implant in this case has demonstrated that the fixation is weakest at the implant‐cement interface. The FE model indicated stresses exceeding the strength of a smooth implant‐cement interface.

Clinical studies have similarly shown loosening at the inferior part of the fixation.^6^, ^7^, ^8^ One study by Nyffeler et al.^20^ found a retrieved loosened glenoid had clearly failed at the implant‐cement interface, however, most studies (with few retrieved glenoids) indicate failure at the cement‐bone interface. The question is why is the implant‐cement interface not observed as loosening clinically?

Although the causes of failure were primarily found at the implant‐cement interface inferiorly, the problem of bone compression, found in a third of the specimens in this study, will also have a long‐term effect on bone remodeling. One of the drawbacks in this testing method is that the mechanobiological element is completely eliminated from the fatigue test. The results in this study suggest that improving the mechanical fixation of the glenoid implant at the implant‐cement interface may improve the short to mid‐term outcomes of the implant. However, the biological effects will inevitably be one of the primary concerns in long‐term outcomes of the fixation. It is at this point that the cement‐bone interface, initially an excellent mechanical interlocking mechanism, may biologically break down into a fibrocartilage‐cement interface. This fibrocartilage layer may be the cause of the progressive radiolucent lines found in radiographs.^21^ It is therefore understandable that early static images of the shoulder do not reveal gaps in the implant‐cement interface, which would manifest under dynamic movement. In a recent radiographic study, Fox et al. highlighted late radiographic failure occurring after 5 years and called for the need for design innovations to improve glenoid fixations.^3^ The study also highlighted glenoid implants “at risk” of radiographic failure were linked with superior subluxation of the humeral head, which may indicate the problem of high vertical head displacement, a measure used in this paper. This is further supported by a fluoroscopic study showing higher superior humeral head migration under dynamic movement compared to static, indicating an underestimation of true head migration during movement.^22^ These findings are possibly supported by an earlier multicenter study in 2002 that among the complications, five shoulders suffered from postoperative humeral head subluxation/dislocation, from which three were due to glenoid loosening and one due to poor rotator cuff support.^23^

Partial implant embedding superiorly was observed in six cases during testing, however, the cross‐sections did not reveal obvious bone crushing in all of them. Despite this, embedding affects the subluxation mechanics, possibly exaggerating further the “rocking horse effect.” Thus, if the implant can avoid embedding into the bone the stability and longevity of the fixation will improve. It may simply be a question of implant seating and correct sizing of the implant to align the implant rim with the cortical glenoid rim as also suggested by Iannotti et al.^24^ Maintaining the subchondral plate is also important to maintain good glenoid seating. However, this study shows radial mismatch does not appear to be critical, which is supported by previous cadaveric and clinical studies.^25^, ^26^

There are several drawbacks in this study; firstly, the rim displacements were often difficult to monitor, due to the compliance of the implant polymer. A stronger correlation to CT and visual failure was found when monitoring failure using vertical head displacement compared to inferior rim displacement. Unfortunately due to the relatively few CT data points for each specimen, it was not possible to identify whether the changes in displacements were directly a result of or preceding failure. More CT scans would be necessary for this analysis. Vertical head displacement best matched visual failure, although this match was not as close as expected. Interestingly, the increased vertical head displacement preceded visual failure in some cases. This supports the “rocking horse” effect explanation, where increasing head translation leads to fixation failure.

The loading regime was displacement controlled and was adjusted every 4000 cycles to maintain consistent loads throughout the tests. Although this would have impacted on the number of cycles to failure, it was necessary to ensure the progression of failure was captured. If tested under load control there was a greater risk that the stages of failure would not be captured, which was an important objective in the study. Despite this limitation, the outcomes on cycles to failure were not intended as directly equivalent to clinical failure and therefore was not set up to test how long the fixation would last clinically.

The third drawback is that only one implant design was tested, thus comments regarding design weaknesses and stress raisers are limited to the particular design. However, restricting the test to one design allowed observations on generic parameters to be made such as the apparent weakness of the implant‐cement strength using a smooth implant. Although using a smooth implant inevitably weakened the interface, this worst‐case scenario is useful to analyze and the most clinically relevant as all companies, with one exception, do not roughen the glenoid implant for cemented TSA.

The finite element analysis used to evaluate the stress/strain behavior was limited to one specimen. Although this limits the discussion on the internal loading behavior, all specimens demonstrated similar failure modes and the FE analysis did corroborate the experimental findings of inferior tensile stresses and implant embedding superiorly in the shoulder. Therefore the similarity in failure behaviors between samples gives some weight to the FE analysis being representative of the general loading trend. However a more detailed investigation on the minor distinctions between samples from experimental observations may still benefit from individual FE analysis.

Finally, a cadaveric study of 10 scapulae is a small one. Variability in bone quality, properties and various implant sizes, resulting in variable radial mismatches, makes conclusive remarks more difficult. In addition, clinical loosening may be affected by biological processes over time, which do not occur with cadaveric tests. This has meant the results in the study may not hold the power needed to conclusively state the strength of using CT images for measuring loosening progression. A post‐hoc power analysis indicates over 60% power (*α* = 0.05). However it does corroborate the glenoid implant bone substitute study that showed a link between interface failure using CT images and actual failure after cutting the samples.^15^ The outcomes also highlight a possible alternative to radiographs that is more informative than other methods on the state of the glenoid interface. Furthermore the positive correlation between quantitative measures and failure were found to be consistent in all samples and also corroborated previous studies.^13^, ^14^ Despite these limitations, testing the cadavers to failure in vitro has allowed valuable insight into the mechanics of the cemented fixation and the various parameters that contribute to the failure of the fixation.

Most clinical studies use radiographs, a common practice to assess the extent of loosening. However, CT has been shown to be better at predicting loosening.^8^ Aliabadi et al.^27^ found no correlation between radiolucent lines around the glenoid in radiographs and pain, function and range of motion. Similarly Yian et al.^8^ found no correlation between radiolucent lines observed on plane radiographs and pain, however, a correlation was found between radiolucent lines observed in CT and pain. Likewise, Nagels et al.^7^ found, using RSA techniques to monitor glenoid motion and loosening, that RSA was better at detecting glenoid loosening compared to radiographs. The non‐specificity of radiolucent lines in detecting loosening and joint function is discussed further by Kovacevic et al.^28^ Thus, although radiographs have been useful to analyse grossly loose implants, monitoring early signs of failure is hit and miss. Gregory et al. further demonstrated the superiority of CT over radiographs, showing CT failure correlating to observed failure of glenoid implants in bone substitute. This paper reports on the first cadaveric study to show a correlation between actual failure progression in vitro to failure observed on CT images and further correlates the in vitro quantitative measures to CT failure.

The issue of substantial radiation dose from CT scans compared to radiographs and subsequently patient safety is a concern. Therefore using CT imaging in its current form may be more suited for more critical cases. However, there is ongoing research and discussion on optimizing CT parameters to minimize dosage and achieve the required image accuracy.^29^ There may be some way to go to find a practical solution to the problem of detecting implant loosening clinically. Despite this the outcomes of this study sheds some light into understanding mechanical loosening. Furthermore the use of CT scanning for implant testing and design development is useful and a more clinically relevant measure of loosening.

## CONCLUSION

Inferior rim displacement and vertical head displacement were both shown to correlate to progressive failure in vitro. Monitoring rim displacement is technically more difficult to implement, highlighting the shortcomings of using this method. Vertical head displacement overcomes this problem. Both measures were found to correlate to visualization of interface failure in CT scans, highlighting the possible usefulness of assessing failure from CT images in clinical practice.

Comparative study of various glenoid designs will require a large sample size, which is unobtainable in a cadaveric study. For such a study the use of a bone substitute with reliable properties is desirable. This study will therefore be an important validation step for investigating design parameters in commercial implants using bone substitute foam as the substrate.

## AUTHORS' CONTRIBUTIONS

S.J.: Primary contribution to research design, the acquisition, analysis and interpretation of data, drafting and revising the paper. T.G.: Substantial contributions to the acquisition and interpretation of data and editing the paper. S.F.: Substantial contributions to the acquisition and analysis of the data. R.E.: Substantial contributions to research design and interpretation of data. A.A.: Substantial contributions to interpretation of data and editing the paper. U.H.: Substantial contributions to research design, analysis and interpretation of data and editing the paper.
